# Is COVID-19 the end of US hegemony? Public bads, leadership failures and monetary hegemony

**DOI:** 10.1093/ia/iiaa134

**Published:** 2020-09-01

**Authors:** Carla Norrlöf

**Keywords:** Covid-19, Liberal democracy, liberal international order, Great Powers, monetary hegemony, hegemony

## Abstract

COVID-19 is the most invasive global crisis in the postwar era, jeopardizing all dimensions of human activity. By theorizing COVID-19 as a public bad, I shed light on one of the great debates of the twentieth and twenty-first centuries regarding the relationship between the United States and liberal international order (LIO). Conceptualizing the pandemic as a public bad, I analyze its consequences for US hegemony. Unlike other international public bads and many of the most important public goods that make up the LIO, the COVID-19 public bad not only has some degree of rivalry but can be made partially excludable, transforming it into more of a club good. Domestically, I demonstrate how the failure to effectively manage the COVID-19 public bad has compromised America's ability to secure the health of its citizens and the domestic economy, the very foundations for its international leadership. These failures jeopardize US provision of other global public goods. Internationally, I show how the US has already used the crisis strategically to reinforce its opposition to free international movement while abandoning the primary international institution tasked with fighting the public bad, the World Health Organization (WHO). While the only area where the United States has exercised leadership is in the monetary sphere, I argue this feat is more consequential for maintaining hegemony. However, even monetary hegemony could be at risk if the pandemic continues to be mismanaged.

COVID-19 jeopardizes all dimensions of human activity, qualifying as the most invasive global crisis of the postwar era. Is COVID-19 the final nail in the coffin for US hegemony? By theorizing COVID-19 as a ‘public bad’, I shed light on one of the great debates of the twentieth and twenty-first centuries regarding the conditions for the decline of US hegemony and its relationship to collective action, international institutions and the liberal international order (LIO).

COVID-19 threatens US hegemony in terms of both US capabilities and US leadership, which it for the most part abdicated during the crisis. Before Trump took office, many scholars and policy-makers believed we were either already in a multipolar world or inexorably moving towards one.^1^ Reasons for these declinist predictions vary. Reverberations from the 2007 financial crisis;^2^ the rise of emerging powers and ‘the rest’;^3^ the relative decline of western values;^4^ the failure of ‘liberal hegemony’:^5^ all have been offered as causes of the coming multipolar order. Militarily, great power resurgence has narrowed the gap between the United States and those on the next level in the global distribution of power, China and Russia. But in terms of raw power projection, America undeniably remains in a league of its own.^6^ Power diffusion has been greater in the economic sphere, where emerging powers—particularly China, India and Brazil—are more competitive.^7^ This is especially true with regard to traditional metrics used to gauge commercial significance, such as export capacity and to a lesser extent import penetration. In terms of more relevant forms of economic prowess—financial power, currency power and its derivative monetary power—the United States continues to excel even as competitors make significant inroads.^8^ The United States also has unprecedented security and economic networks, which can be leveraged to its advantage to maintain pre-eminence.^9^

Even before COVID-19, however, the Trump presidency was undermining US hegemony.^10^ The networked relations underpinning that hegemony have been strained. Trump's questioning of US security alliances, the trade order, the dollar system and climate agreements has weakened the US-led order. Should he win another presidential term, and continue to alienate America's security and economic allies, states may seek to develop serious alternatives to US power despite the costs they would initially face in doing so. Any credible alternative to US power would require a strong show of force, both militarily and economically, and the replacement of US financial infrastructure. Challengers would have to strike at the heart of US power: to supplant US military dominance and security relationships, unseat the dollar as the global currency and build a dominant financial base. US power may continue to decline relative to other powers, but in the current system absolute decline is unlikely unless a substitute power emerges.

Is COVID-19 the crisis that will eclipse US hegemony? US hegemony, which requires not just dominance but leadership, has already eroded somewhat. During the pandemic, US leadership has been restricted to monetary leadership. US failure to exercise leadership in other areas presents real risks for its hegemony because unlike other international challenges, the US has limited opportunities to externalize the costs of the public health crisis. However, to date no alternative to US hegemony has emerged: the pandemic complicates the realization of a liberal alternative to the United States, and also makes a Chinese or Russian alternative less likely because these states too have been primary sites of the pandemic and pursued delegitimizing policies in the process.

The article is organized as follows. First, I characterize the COVID-19 crisis as a public bad. I then discuss the United States’ domestic and international response to the health crisis. Domestically, I argue that failure to effectively manage the COVID-19 public bad has compromised America's ability to secure the health of its citizens and the domestic economy, the very foundations for its international leadership. Internationally, the US has used the crisis strategically to reinforce its opposition to free international movement while abandoning the primary international institution tasked with fighting the public bad, the World Health Organization (WHO). The only area where the United States has exercised leadership is in the monetary sphere, to stabilize the international economy. Powered by US dollar hegemony, the least institutionalized component of the LIO, the Federal Reserve has thus served as global lender of last resort. In concluding, I predict the effects of COVID-19 by evaluating the likelihood that US failure to manage this public bad may undermine other public goods and advance other centres of power at the expense of US hegemony.

## COVID-19 as a public bad

This section maps the global spread of the disease and clarifies what we know about the virus, its deadliness and its spread across regime types. I conceptualize COVID-19 as a public bad and contrast this abstraction with the public goods assumption upon which theories of hegemony are based.

### The global spread of COVID-19

A new coronavirus, SARS-CoV-2, causes a severe acute respiratory illness, COVID-19. Humans infected with the virus show varying symptoms. Some individuals report fever, a dry cough, sore throat, fatigue and body pain. Others display mild symptoms such as loss of smell and taste. Yet others remain entirely asymptomatic. Surgical facial masks seem likely to prevent COVID-19 transmission from symptomatic individuals.^11^ COVID-19 can be fatal. The symptomatic case fatality rate was estimated to lie around 2 per cent in Wuhan, the Chinese province where the infection began to circulate.^12^ The overall case fatality ratio has been estimated to be higher outside China at 2.7 per cent.^13^ Preliminary research suggests that individuals belonging to blood group A face a higher risk of COVID-19 infection than those of other blood groups, and that individuals belonging to blood group O face a lower risk of COVID-19 infection than other blood groups.^14^ Symptomatic infections and the rate of case fatalities increase with age, particularly in those aged over 59 years.^15^

COVID-19 is a global phenomenon: as of July 2020, there are only a dozen countries reporting no cases. To date, 13 million cases have been declared worldwide with nearly 600,000 deaths.

*The COVID-19 black box* Divergent demographics as well as data limitations explain some of the country differences in disclosed case fatality rates. As the pandemic continues to evolve, so its incidence and death toll across countries continue to shift. Today's hotbeds may turn out to be more similar to other countries, in terms of overall cases and case fatality rates, than they now appear to be. The available COVID-19 data suffer from considerable selection bias, complicating strong inferences. Three main problems make country assessments and inter-country comparisons difficult.

First, countries vary considerably in terms of the resources they have available to collect data, their reporting capacity and their transparency. Both the degree of accuracy and the sincerity of the reporting are likely to vary across countries.

Second, inadequate testing creates an inherent bias, since we do not know the universe of cases. Two types of test exist. One type of test establishes whether a person has been infected with the virus. A second type tests for immunity. This second antibody test, also known as a serological test or sero-survey, determines whether infected people have developed antibodies against the virus. The first test is the most direct way to gather information about the number of infected people. The second test also provides information about the number of infected people, because those who have developed antibodies are presumed to have contracted the virus before developing antibodies. The primary purpose of the second type of test, however, is to assess what portion of the population can be safely reintegrated into society and resume normal activities.

Widespread testing is desirable, although testing a smaller share of the population might accurately reflect the true number of cases if randomized. Most countries have had resources only to test people with serious symptoms. That is a major problem, given the evidence from China suggesting that 78 per cent of new COVID-19 infections are asymptomatic.^16^ So far, very few countries have conducted random nationwide testing. Without large-scale testing or random testing, there is no way of knowing the true number of cases. The higher a country's known rate of infection, the greater the problem posed by inadequate testing, given the multiplicative spread of the disease. Some countries have started rolling out sero-surveys. However, the reliability of the antibody tests is in dispute: first, because it is possible that they confuse COVID-19 with other illnesses, and, second, because people who have developed antibodies may not actually be immune to COVID-19. Like the first type of COVID-19 test, the antibody test should be performed through random sampling in order to reach more reliable estimates of both the actual proportion of the population that is infected and the proportion that can safely emerge from isolation. Without universal, or random, testing it is impossible to know the ramifications of the pandemic in terms of its extent or deadliness. Even if testing is randomized, it remains difficult to know the case fatality rate since we cannot know how many untested people have died from COVID-19.

The case fatality rate and deadliness of the disease are also influenced by demographic factors. Everything else being equal, we should expect countries with older populations to have a higher case fatality rate. That is because older people infected with SARS-CoV-2 face both a greater risk of dying from COVID-19 complications and a greater risk of dying even though COVID-19 was not the immediate cause of death.

A third problem arises because countries vary in terms of what they report. Some countries automatically count a deceased person infected with SARS-CoV-2 as a COVID-19 death even though no effort was made to establish whether it was the actual cause of death. Other countries, such as the United States, base numbers of COVID-19 deaths on death certificates, and therefore rely heavily on the judgement of the treating doctor or pathologist. Some countries report only those COVID-19 deaths that occur in hospitals, not those in private or retirement homes.

While we are in a continuously evolving and therefore fluid situation, an analysis of the virus's trajectory in its initial phase is useful in studying governments’ responses to the outbreak—particularly the responsiveness of the world's hegemon, the United States, and other liberal democracies supporting the LIO.

*Is COVID-19 a liberal curse?* In this section I discuss the relationship between liberal democracy, cases per capita and case fatalities, using two different measures of liberal democracy. I do this for two reasons. First, I seek to determine whether open societies are more vulnerable to the pandemic. Second, if liberal democracies are indeed hit harder than other regime types, a liberal alternative to US hegemony will be difficult to achieve.

**Figure 1:**
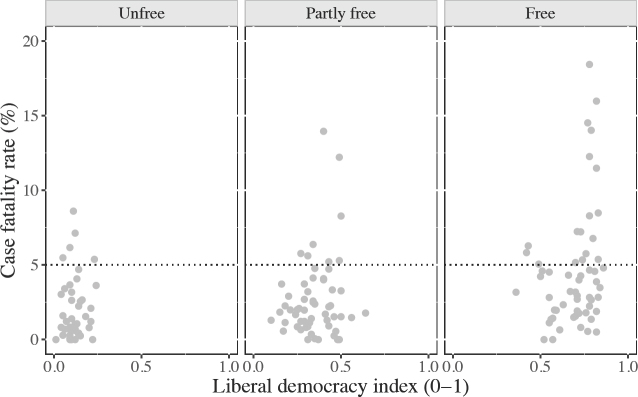
Case fatality rates and freedom levels

*Notes*: A horizontal dotted line is placed at a case fatality rate of 5%. The categories are based on Freedom House types and the X-axis is based on the degree of liberal democracy according to the continuous V-Dem variable, v2x_libdem.

*Source*: Author's calculations based on European Centre for Disease Prevention and Control, *Covid-19 geographic distribution worldwide 2020-05-03* (Solna, 2020); World Bank, *World Development Indicators: population, total* (Washington DC, 2020); Freedom House, *Global Freedom Scores* (Washington DC, 2020); Michael Coppedge et al., *V-Dem Dataset V10* (Gothenburg: Varieties of Democracy (V-Dem) Project, 2020).

COVID-19 initially spread rapidly within liberal societies, though early measures were taken to slow its spread in most countries, except for the United States. Based on Freedom House's taxonomy, figure 1 associates countries’ case fatality rates with freedom levels. Freedom House's global freedom score contrasts ‘free’ countries with countries which are either ‘partly free’ or ‘not free’ (labelled ‘Unfree’ in the figure). The majority of countries with fatality rates above 5 per cent by late June 2020 are labelled ‘free’ by Freedom House.^17^

The global score encompasses both political rights and civil liberties and therefore serves as a composite proxy for liberal democracy. According to this measure, a liberal democracy protects citizens’ political rights, property rights and civil rights. While the global freedom score for the most part corresponds with what counts as a liberal democracy, some exceptions exist. For example, Monaco is a constitutional monarchy and not a liberal democracy, yet Monaco is characterized as ‘free’ according to the global freedom score. I also use the V-Dem score to evaluate the degree of liberal democracy on a continuous scale.^18^

As noted above, there are many good reasons to be cautious about the reliability and comparability of the underlying data, and therefore to exercise some caution with respect to the ‘liberal curse’ argument. Statistics from unfree or authoritarian regimes may be more biased than statistics from liberal democracies. Again as already noted, countries have different procedures for determining whether a death was caused by the SARS-CoV-2 virus; some countries, notably Russia, have an especially high bar for counting COVID-19 as the cause of death. The political costs of erroneous or insincere reporting are also likely to be higher in liberal democracies than in other countries, given the electorate's ability to sanction bad behaviour at the ballot box. For all these reasons, it is easy to dismiss the possibility of a deadly disease such as COVID-19 spreading more easily and with greater deadliness in liberal democracies than in other countries.

While it is possible that what appears to be a liberal democratic curse really reflects superior statistics and reporting practices in liberal democracies, there is at least a possibility that the embeddedness of liberal democracies in an interdependent and interconnected world characterized by relatively free cross-border flows of goods, services, assets and people has accelerated and amplified the virulent effects of the disease. We should expect people residing in open, internationally integrated societies to face a higher risk of exposure, making such societies more susceptible to viral diffusion. As is clearly visible in figure 1, case fatality rates increase with freedom ratings and as we move towards more ‘free’ regimes on the X-axis.

**Figure 2:**
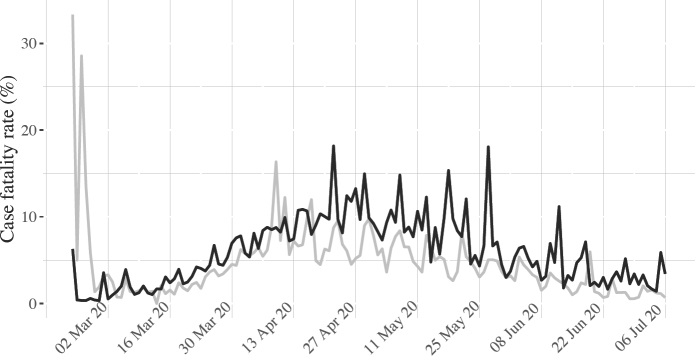
Average case fatality rates in the United States and in other liberal democracies

*Note*: The grey line is the case fatality rate in the United States; the solid black line is the average case fatality rate in other liberal democracies.

*Source*: Author's calculations based on European Centre for Disease Prevention and Control, *Covid-19 geographic distribution worldwide 2020-05-03* (Solna, 2020); Freedom House, *Global Freedom Scores* (Washington DC, 2020).

Over time, the case fatality rates in the United States and other liberal democracies have converged, but the rate remains quite a bit lower in the United States, with fewer sharp peaks (see figure 2). With the recent explosion in US cases, however, US deaths are bound to escalate. Given the evolution in other liberal democracies, we may very well see the US line rise above the other line in figure 2 towards the end of the summer and as we enter autumn.

**Figure 3:**
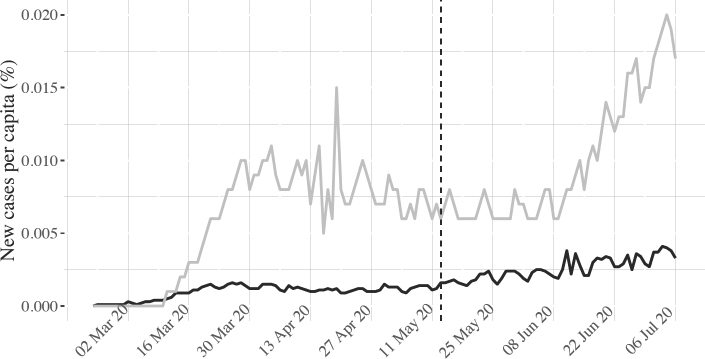
Per capita new cases in the United States and in other liberal democracies

*Notes*: The grey line shows the percentage of new cases per capita in the United States; the solid black line shows the percentage of new cases per capita in other liberal democracies. The vertical dashed line at 20 May 2020 marks the phased lifting of lockdown restrictions in the United States.

Figure 3 compares the evolution of new cases per capita in the United States and in other liberal democracies. US cases are clearly growing more strongly. By July, new US cases were equivalent to 1.7 per cent of the population, whereas the corresponding figure across liberal democracies was only 0.1 per cent.

The inability to curb the spread of new cases points to failed leadership in the United States compared to other liberal democracies. While the comparison may speak to the fact that the outbreak in other liberal democracies came earlier, the premature reopening of the US economy and public spaces in many states on 20 May 2020 (indicated by the dashed vertical line in figure 3) is likely to be a major reason for the subsequent upward surge. While new cases remained rather steady in liberal democracies, US cases started to explode within a fortnight of restrictions being lifted. Yet, sadly, this is only one aspect of the flawed US strategy, discussed further below.

### Conceptualizing COVID-19 as a public bad

Conceptualizing COVID-19 as a public bad has leadership implications. Public goods and public bads share two properties. The first property, jointness of supply, means that once supplied a state's enjoyment of the public good does not preclude its enjoyment by other states. The second property, non-exclusion, means no state can be prevented from enjoying the good, regardless of contributions to them.

The specificity of the COVID-19 public bad is usefully illustrated by an analogy with climate change prevention. If we think of clean air as a global public good, one state's enjoyment of the good does not interfere with another state's enjoyment, and no state can be excluded from enjoying clean air. However, state efforts to mitigate climate change are best understood as an effort to prevent the public bad, resulting in some rivalry when states shirk preventive efforts. More specifically, preventive measures consist in reducing greenhouse gas emissions, requiring either lower or modified output.^19^ States which choose to prevent the public bad therefore prioritize the environment at some economic cost. A state which chooses to prioritize the economy fails to prevent the public bad. Failure to combat the public bad negatively impacts other states as they become exposed to more of the public bad as well as to the costs of competing with the non-abating state (as reflected by relatively more expensive output and exports).

According to the second property, climate change mitigation is non-excludable: no state can prevent climate change (or economic) harms from states which do not participate in preventing the public bad.

The presence of some rivalry, without any possibility of exclusion, implies that states which do not practise global climate mitigation can free ride environmentally and economically on states which do. Even if large states, such as the US, eventually hurt themselves in the process of hurting others by not mitigating, they can shift a large part of the mitigation burden onto other states precisely because climate change mitigation combines some degree of rivalry with non-excludability.

COVID-19 can also be characterized as a public bad, it is non-rival since being infected does not prevent another person from being infected. COVID-19 is also non-excludable because in the absence of a vaccine, no one can eliminate the possibility of being infected. As with climate change, we can also conceptualize the international effort to mitigate COVID-19 as a public bad. According to the first property, some rivalry is present. When states seek to combat COVID-19 through lockdowns and other safety measures, they reduce output and trade. Differently from the climate change public bad, the extent of rivalry with regard to the COVID-19 public bad is, however, contingent on other international public goods, most notably open borders but also free trade. Under conditions of openness, states eschewing efforts to prevent the public bad negatively impact other states’ ability to prevent the COVID-19 public bad. In an open world, states face both public health and economic rivalry, as in the climate change example.

However, according to the second property, and differently from the climate change example, states can exclude one another from the health portion of the public bad by closing borders. The ability to limit international human mobility implies the absence of public health rivalry, though some degree of rivalry nonetheless persists due to economic rivalry. No opportunities exist for free riding on other states’ public health. Opportunities only exist to free ride on other states’ restrained economic activity. This has wide-reaching implications because no state can induce other states to bear a greater overall public bad burden by externalizing failed public bad prevention. States can shift the economic burden of the pandemic onto other states, but not the public health burden. US failure to adequately address the COVID-19 public bad presents a health crisis for the US without any public health incidence on other states because public bad prevention can be made partially excludable, transforming it into something in between a public and a club bad.

The failure to exercise leadership in preventing the COVID-19 public bad poses greater risks than the failure of leadership in providing other public bads or goods. In addition, failed public bad prevention is very likely to threaten other public goods, whether accidentally or by design. The US administration's failure to adequately manage the public bad has real consequences for securing the health of its citizens and its economy, potentially jeopardizing its capacity to use its hegemonic position to provide public goods. At the same time, the public bad has been used strategically to undermine the US commitment to relatively open borders.

## Hegemony and the COVID-19 pandemic

In this section I discuss the domestic and international engagement of the US administration during the first six months of the pandemic in order to assess what it has done to prevent the COVID-19 public bad. Crisis times are precisely when the dominant actor within the order, the hegemon, is expected to behave responsibly and organize states to solve collective action problems. Hegemonic stability theory and theories of hierarchy lead us to expect the hegemonic actor to mitigate the hazardous long-term effects of COVID-19 on other states and on the public goods that define the LIO.

Public good provision and public bad prevention require strong domestic foundations. Securing the health of American citizens is vital for domestic economic stability, even given the existence of short-term trade-offs between securing the health of Americans and that of the economy. While the United States has expended enormous resources on keeping Americans safe from external military aggression, it has spent far less on generalized health care and social safety nets. This failure to provide domestic public goods has created long-term fissures between winners and losers from globalization, putting hegemony at risk long before the pandemic arrived on US shores.^20^ The under-provision of domestic public goods and the retreat from ‘embedded liberalism’—whereby the benefits from openness are redistributed through greater safeguards and labour adjustment programmes—did not originate with the Trump administration.^21^ But the administration intensified these trends in the area of health by reducing funding for the Centers for Disease Control and Prevention (CDC). By negating the science pertaining to the virus, and by responding slowly and inadequately, the government has prolonged rather than contained the public bad, further undermining the domestic foundations of its hegemony.

As for the administration's international response, it has been fragmented. Instead of organizing states to fight the ‘public bad’, the Trump administration used the crisis to crack down on open borders and threatened to rescind financial contributions to the WHO, eventually promising to withdraw from the institution altogether with effect from 1 July 2021. As the United States has become a focal point of the crisis, US leadership has been restricted to the exercise of monetary hegemony.

### Undermining US legitimacy

Up to mid-March 2020, President Trump denied the scope, gravity and lethality of COVID-19, ignoring warnings from his own administration, US intelligence and the WHO.

The White House received its first reports of the virus on 3 January 2020, based on talks between the director of the CDC and its Chinese counterparts.^22^ Throughout January and February, US intelligence agencies issued dire warnings, even raising the possibility that Chinese officials were minimizing the scope and danger of the epidemic.^23^ On 30 January, the WHO declared the COVID-19 pandemic a Public Health Emergency of International Concern.^24^ The organization warned all countries to ‘be prepared for containment, including active surveillance, early detection, isolation and case management, contact tracing and prevention’.^25^

On 21 January, a day after the first COVID-19 case was reported in Seattle, President Trump said: ‘We have it totally under control … It's going to be just fine.’^26^ As early as 29 January, White House trade adviser Peter Navarro issued a memo warning of the ‘risk of the coronavirus evolving into a full-blown pandemic, imperilling the lives of millions of Americans’.^27^ He is also believed to be behind a memo to the president on 23 February, advising the government to scale up investments in ventilators and personal protective equipment (PPE) for medical personnel to the tune of $618 million, owing to the ‘increasing probability of a full-blown COVID-19 pandemic’.^28^

On 14 February, the US Department of Health and Human Services (HHS) drafted a joint memo with the National Security Council recommending substantial limitations to public gatherings to stop the spread of the disease.^29^ One week later, the White House coronavirus task force, along with Dr Anthony S. Fauci of the National Institutes of Health and Dr Robert R. Redfield of the CDC, generated flu pandemic scenarios and concluded that complete social distancing would be necessary to manage the crisis.^30^ Around the same time, the WHO warned of a potential pandemic following a significant spike in cases in Lombardy (Italy) and Iran. Nancy Messonnier, the director of the National Center for Immunization and Respiratory Diseases, made a public statement regarding the inevitability of the pandemic afflicting the United States, urging citizens to take appropriate measures.^31^ President Trump expressed public scepticism and emphasized the ‘low level’ of cases and exceptional recovery rate in the United States, concluding: ‘I don't think it's inevitable.’^32^ Seeking to reassure the public, he affirmed: ‘Whatever happens, we're totally prepared’—but declined to be specific about the measures required to contain and stop the spread of the virus, thus creating the opposite effect.^33^ At the end of the month, the United States faced its first known COVID-19 death.

On 11 March, the WHO declared a global pandemic. However, it was not until 13 March that President Trump declared a national emergency, granting emergency authority to the Secretary of the HHS,^34^ and extra authority to the Secretary of the Treasury to respond to the crisis.^35^ Despite early warnings, attempts to minimize the health scare persisted until mid-March. On 16 March, the president issued ‘Coronavirus Guidelines for America’ advising Americans to follow social distancing and hygiene practices.^36^

Besides being slow to respond to the crisis, the US administration also sought to undermine the scientific consensus on the origins of SARS-CoV-2, putting pressure on US intelligence to support unproven theories of the virus's origins in a Wuhan laboratory.^37^ The Wuhan lab theory conflicts with the scientific consensus, which suggests COVID-19 originated in bats.^38^ Scientists have debated three transmission mechanisms—zoonotic transfer following natural selection in an animal host; natural selection in humans following zoonotic transfer; and selection during laboratory escape.^39^ While there is no consensus as to whether the virus had already mutated before infecting humans or if it mutated after infecting humans, consistent with the second mechanism, scientists agree on the implausibility of the virus originating in a lab.^40^ ‘The genetic data irrefutably show that SARS-CoV-2 is not derived from any previously used virus backbone.’^41^

By ignoring all the signs of a health crisis of enormous proportions and engaging in science denial, the United States has dealt a huge blow to its own legitimacy.

### Sluggish, self-defeating domestic engagement

The economic ramifications of the crisis have been extraordinary, surpassing the calamity wrought by the 2007–2008 financial crisis in several respects. Below, I discuss the astounding dislocation caused by the pandemic and the extraordinary steps the United States has taken to mitigate the economic downturn. Despite these unusual steps, the sluggishness of both the response and its implementation, as well as underlying inequities in access to health care, have combined to exacerbate the damage caused by the public bad, leaving the domestic foundations of US hegemony hanging in the balance.

**Figure 4:**
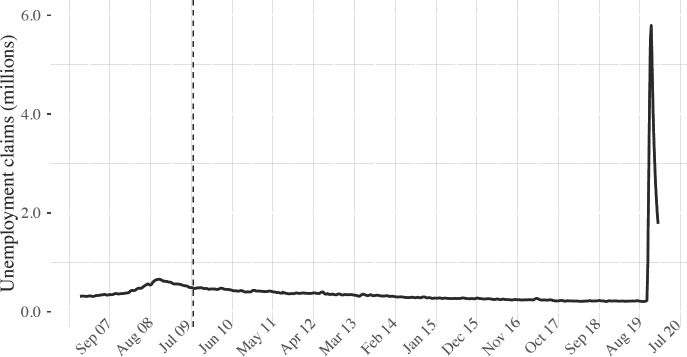
Four-week moving average of US unemployment claims, September 2009 to July 2020

*Note*: The vertical dashed line at December 2009 marks the end of the financial crisis.

*Source*: Author's calculations based on seasonally adjusted US Labor Department data.

**Figure 5:**
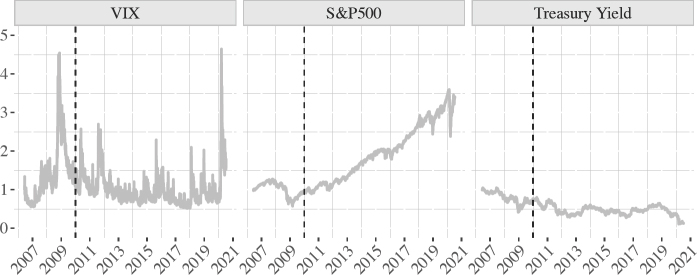
Indexed financial indicators, June 2006 to July 2020 (June 2006 = 1)

*Note*: VIX is the Volatility Index of the Chicago Board Options Exchange (CBOE).

Downward pressure on household incomes and savings is to be expected during a pandemic of this magnitude, further reducing demand and generating lay-offs. To put the economic calamity into perspective, figure 4 shows rolling averages of US unemployment claims, an initial sign of job losses. A dashed vertical line is placed at the end of December 2009 in figure 4 (and figure 5 below) for easy comparison with the economic perturbations caused by the 2007–2008 financial crisis. Figure 4 shows starkly that unemployment claims filed as a result of the pandemic vastly exceed those filed during the financial crisis. Since the first US-based COVID-19 death was reported in late February, over 50 million Americans have filed for unemployment benefits. We now have a debt-for-equity trap in the making with particularly devastating consequences for households financing property acquisition via short-term rentals, equity returns and dividends. Defaults on US mortgage payments rose by over 120 per cent between March and May 2020.^42^

The vertical dashed line at December 2009 marks the end of the financial crisis.

*Source*: Author's calculations based on figures from Global Financial Data (San Juan Capistrano, CA, 2020).

Key financial indicators are indexed in figure 5 for easy comparison of changes in financial volatility, stock market performance and Treasury yields relative to the summer of 2006, preceding the global financial crisis. As the first panel shows, fear in global markets peaked in mid-March 2020, surpassing the severe market reaction during the 2008 financial crisis. As is visible from the second and third panels, March 2020 also saw steep falls in the world's major stock markets and in US Treasury yields. While stock markets have rebounded, the real economy continues to suffer, and the Federal Reserve predicts unemployment to reach 7.5 per cent by the end of the year.^43^ The fall in the Treasury yield curve offers yet more confirmation of the dismal circumstances, while also suggesting a perk for the US government in the form of lower borrowing costs.

The implicit rebate on US borrowing provides the Treasury with greater leeway to make use of the additional authority bestowed upon it by President Trump. On 25 March, the Senate passed the Coronavirus Aid, Relief, and Economic Security (CARES) Act in a bipartisan vote, offering relief to households and businesses. The relief package has four components: Assistance for American Workers and Families; Small Business Assistance (SBA);^44^ Preserving Jobs for American Industry; and Assistance for State and Local Governments.^45^ So far, these funds have been disproportionately allocated to businesses (44 per cent), although a portion of their package is designed to help workers; 29 per cent is available for individuals, with the remaining 27 per cent destined for government, including programmes for health care and education. On 21 April, the Senate passed a new economic relief package to the tune of $484 billion.^46^

Despite the unprecedented ambition of these rescue packages, cracks quickly appeared in their different components. On 16 April, the SBA ran out of money for its Payroll Protection Program.^47^ Even before funds ran out, multiple issues, ranging from technological glitches to complex application procedures and inequitable distribution of funds, plagued its implementation. At the same time, overwhelmed unemployment offices have struggled to disburse assistance to the 22 million Americans seeking unemployment benefits.

The Federal Reserve also stepped in to prop up the economy. Zero rates of interest were announced on 15 March: specifically, a downward adjustment to the federal funds rate to a band between 0 and 0.25 per cent in support of employment and price stability.^48^ To ensure the smooth functioning of the economy and continued credit flows to businesses and individuals, the Federal Reserve has committed to an indefinite increase in its holdings of Treasury securities and agency mortgage-backed securities, including agency commercial mortgage-backed securities.^49^ New programmes to the value of $300 billion make financing available to businesses and individuals through a number of arrangements, such as funds, credit and loan facilities supporting new bond issues, while also offering liquidity support for outstanding bonds.^50^ The Main Street Business Lending Program promotes lending to eligible small and medium-sized businesses and is intended to complement provisions made under the SBA.^51^

As noted above, the unequal distribution of income and wealth in the United States aggravates the challenges posed by the pandemic. While inequality has not intensified under the Trump presidency, fewer Americans now have health coverage.^52^ Over 28 million Americans had no health insurance in 2018, representing an 8 per cent increase from 2017.^53^ For obvious reasons, the high number of uninsured Americans is of particular concern during a pandemic. Meanwhile, the Trump administration has taken several conscious decisions to enact social welfare cuts, notably in health care. The administration's diminution of agencies and departments vital for protecting the health of American citizens includes the downsizing of the CDC mentioned above, closing the global health security unit of the National Security Council, abolishing the government's $30 million Complex Crises Fund and slashing spending on health by $15 billion.^54^ More generally, the president's 2021 budget included ‘$2 trillion in cuts to safety net programs and student loan initiatives’, including Medicaid and food aid.^55^ The Kaiser Family Foundation (KFF) also warns that President Trump's plans to challenge the constitutionality of the individual mandate contained in the Affordable Care Act in the Supreme Court risks undermining the entire bill and many Americans’ ability to remain insured.^56^ The KFF also notes continuous health-care challenges such as non-universal coverage and high deductibles.^57^ The high numbers of Americans who do not have access to health care, cannot afford health care or have to postpone medical care for financial reasons are not only at risk themselves but pose a risk for all Americans.

In its inability to contain the public bad, and to provide public goods to reduce its spread, the US government has failed to secure the health of its citizens and thereby weakened the economic foundations of US hegemony.

### Fragmented international engagement

The administration's fragmented international engagement combines the absence of leadership for global public bad prevention with successful global monetary leadership. The US government has failed to organize collective action in order to combat the public bad; it has used the pandemic strategically to limit openness beyond measures required to protect American citizens; and it has withdrawn funding and participation from the WHO, the main international institution charged with solving the crisis. Meanwhile, it has exercised monetary leadership.

*Tightening immigration rules* Like other countries, the Trump administration responded to the pandemic by restricting entry into the United States. Incoming travel from China was banned on 31 January, but only after three major US airlines (American Airlines, Delta and United) had already suspended flights from China.^58^ On 29 February, restrictions were imposed on entry into the United States via Iran.^59^ It would take longer to restrict travel from allied countries, despite their reporting more cases. Americans were, however, advised not to travel to ‘specific regions in Italy and South Korea’.^60^ Travel from the European Schengen area to the United States was suspended with effect from 14 March. Separate joint statements with Canada and Mexico were made to temporarily restrict all non-essential travel.^61^ These border restrictions were not unusual.

Certain immigration measures, however, recalled the president's election promises regarding the need for stringent ‘economic security’ policies. Even before the pandemic, the US 2021 budget proposed funding to curb immigration. The budget included $2 billion to complete the border wall with Mexico; $182 million in remuneration for more border patrol officers and the construction of processing centres; $544 million in remuneration for immigration and customs enforcement agents and immigration court prosecutors; and $3.1 billion for some 60,000 beds in Immigration and Customs Enforcement detention centres.^62^ In March, the US government issued a public health order allowing for the prompt removal of migrants, intended to curb humanitarian immigration at the southern border. Legal immigration has also been curbed. An immigration proclamation expressing concerns about ‘the impact of foreign workers on the United States labor market’ initially suspended immigration visas for two months, and has now been prolonged until the end of the year.^63^ What is striking about these measures is that they are not aimed at securing public health for Americans but rather at addressing the effects of the pandemic by reinforcing the 2017 National Security Strategy, which sought to reboot US grand strategy by casting ‘economic prosperity as a pillar of national security’.^64^ On 6 July, Immigration and Customs Enforcement announced that foreign students at universities offering online courses would be deported if they did not enrol in a university programme offering in-person courses. Following lawsuits filed by Harvard University and MIT, the government rescinded its decision on 14 July.

*Abandoning international institutions* On 7 April, President Trump said he would ‘put a hold’ on US funding for the WHO, criticizing the organization for its slow crisis response and ‘China-centrism’.^65^ His proposal alarmed US experts and politicians, who highlighted the need for a coordinated global response based on information-sharing and science. The president of the American Medical Association said ‘fighting a global pandemic requires international cooperation and reliance on science and data’.^66^ House Speaker Nancy Pelosi concurred, questioning the president's authority to withdraw funding, and calling his decision illegal and dangerous for ‘ignoring global health experts, disregarding science’.^67^ Because the United States is required to provide a year's notice of withdrawal, and liquidate its financial obligations before doing so, the president's decision of 29 May can only take effect in the summer of 2021. The decision itself could be revoked if the Democratic contender for the presidency, Joe Biden, is elected in November 2020. The size of America's WHO contribution suggests that the organization will be severely hampered in its effort to coordinate a response to the pandemic if President Trump does indeed follow through with his threat to halt US funding. Figures for 2018/19 show that the United States contributes approximately 20 per cent of the WHO's aggregate budget, which consists of both membership dues and voluntary contributions.^68^

How serious is the rift between the United States and the WHO? US withdrawal, on top of its failure to coordinate policy with the WHO, will make the United States more vulnerable to health crises because US health experts will no longer have access to information exchanged within the institution. Combined with the reduction of funding for the CDC, withdrawal from the WHO represents US abdication of leadership in the face of global health crises, and could make it harder for the United States to manage COVID-19 and future public health bads. This raises two subsidiary questions.

First, how central is combating public health bads to US hegemony? As argued in the previous section, the US government's unresponsiveness to the COVID-19 public health bad could erode the foundations of US hegemony by making it more difficult to secure the good health of its citizens and economy.

Second, how central are America's engagement with and leadership of the WHO to the continuity of US hegemony and the LIO? As a multilateral institution, which sets public health norms and monitors their implementation, the WHO qualifies as part of the ‘open and loosely rule based’ order intended to provide ‘a foundation in which states can engage in reciprocity and institutionalised cooperation’ compatible with different levels of hierarchical differentiation.^69^ Hitherto, however, the WHO has not been central to the LIO because a health crisis of current proportions has never been as widespread, protracted or deadly, or touched advanced countries to the same degree, as the present one. US abandonment of the WHO in and of itself signals that the United States no longer wants to play a key role in public health governance, thus necessarily diminishing its already uncertain commitment to the leadership aspect of hegemony.

Three factors limit the impact of the current tensions on US hegemony and the LIO. First, if the incumbent president does not win a second term, the decision might be reversed. Second, the WHO is not highly institutionalized but rather functions as a depository for scientific and policy exchange, and a channel for collaboration with various support functions, to facilitate the voluntary implementation of established public health norms. Third, US support for the WHO has never been central to the former's hegemonic position or to the LIO. The prospective demise of US hegemony, and of the LIO, is highly unlikely to arise from this particular conflict or decision. It is much more likely to arise from the half-hearted and mostly botched attempt to stop the public bad from creating the most serious existential threat, in the form of a health emergency, the United States has experienced short of war, and its ensuing economic damage.

*Leveraging dollar hegemony* As banker to the world, the US Federal Reserve has tremendous power, and its actions affect markets worldwide. The Federal Reserve not only functions as the US central bank, but also acts as ‘lender of last resort’ in times of international crisis. The United States has not always taken on this role. When Charles P. Kindleberger outlined the criteria for avoiding another Great Depression, he based his recommendation on the failure of the Bank of England and the Federal Reserve to stabilize the international economy. The policies he proposed included providing liquidity and counter-cyclical flows of capital.^70^ During the 2008 financial crisis, the Federal Reserve assumed the role of global lender of last resort and is doing it again, on a bigger scale.^71^

During crises, dollar swaps are the bull's eye of concentric rings of dollar activity spreading outward in the financial system. The motivation for these swaps is clear. Their goal is to prop up financial markets by pumping dollar liquidity into foreign central banks, which then pass on dollars to their respective banking systems. Dollars are literally swapped—exchanged for the receiving country's currency. Sending dollars abroad during a pandemic, or a financial crisis, is necessary to quell fear and panic as financial markets suffer huge asset sell-offs (see figure 5). Such times prompt a generalized flight to safety in cash and bonds, most of all dollars and dollar-denominated bonds. The greenback owes its appeal to its disproportionate use in global trade and finance and its unique ‘safe haven’ status in the global economy. Dollar hegemony creates a scramble for dollars in times of crisis. Only the Federal Reserve has the authority to satisfy global demand for dollars by printing more of them.^72^ Dollar emergency lines are thus typically initiated by the Federal Reserve. This type of government influence has a huge impact on market decisions and is a form of structural power.^73^ The exercise of structural power through currency swap programmes was what provided the Federal Reserve with credit-rationing opportunities during the financial crisis.^74^

We now see this form of structural power being passively deployed in the context of the pandemic and combined with more direct attempts at exerting influence that reward allies and sanction challengers. The Federal Reserve is able to do this because it has ultimate authority over who gets emergency dollar funding. Dollar pipelines have been extended to US allies, with the central banks of Canada, the United Kingdom, Japan and Switzerland, along with the European Central Bank, first in line.^75^ Shortly thereafter, nine additional allies entered into swap agreements with the United States.^76^ America's geopolitical rivals Russia, China and Iran have not received dollar lines and are as unlikely to receive them as they were during the 2007–2008 financial crisis.

Swaps signal central bank cooperation and, owing to their ‘strong announcement effect’, have the capacity to alleviate exchange rate pressures.^77^ For example, under the 1960s Bretton Woods swaps, ‘the announcement of an increase in the available credit under a swap line proved as effective in stemming speculative sales of a deficit-country's currency as the actual use of the line’.^78^ Swaps raise the risk and cost of taking speculative actions against a currency, selling it short. They ‘lean against the wind’, directing market participants’ expectations and actions. Indeed, the Federal Reserve's pandemic swaps have eased depreciation pressures for countries which saw their foreign exchange reserves dwindle relative to their dollar-denominated debt, and also for oil and gas exporters such as Canada and Norway which saw their currencies plummet as a result of sagging commodity demand.

On 14 March President Trump threatened to demote the Federal Reserve chair Jerome H. Powell for failing to act aggressively to contain the economic fallout from the pandemic. The following day he applauded the Federal Reserve's actions, notably the swap lines and the move to zero rates. At a press briefing, he said: ‘I'm talking about the Federal Reserve … it's a tremendous thing that took place just now … I'm very happy.’^79^

**Figure 6:**
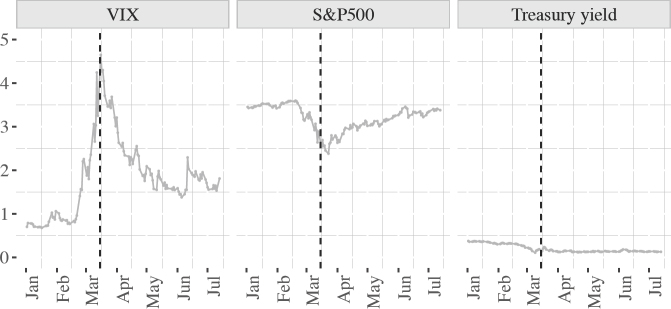
Indexed financial indicators, January 2020 – July 2020 (June 2006 = 1)

*Note*: The vertical dashed line at 15 March 2020 marks the onset of the Federal Reserve's swap lines and zero rates.

*Source*: Author's calculations based on figures from Global Financial Data (San Juan Capistrano, CA, 2020).

So, while the Federal Reserve is technically independent, it is possible that the president's rebuke of 14 March forced the Fed's hand into a show of monetary leadership on 15 March, though these agreements with foreign central banks are likely to have been forthcoming anyway at some point. What precisely caused the timing of the swaps is irrelevant for our purposes. What is relevant in assessing the impact of the pandemic on US hegemony are the unprecedented measures taken by the Federal Reserve to prevent a global economic downturn. Its beneficial effects in containing the calamity in financial markets prior to the 15 March announcement cannot be overstated. The impact is put on display in figure 6, which projects figure 5 across the first seven months of 2020. The first panel shows the dramatic fall in market volatility; the second panel shows the rebound in the S&P500 stock market index after 15 March. The United States has very clearly exercised effective monetary leadership during the pandemic, extending swap agreements and aggressively cutting rates, on a scale surpassing its monetary leadership during the 2007–2008 financial crisis.

Though far less studied than US commercial or security engagement, US monetary leadership is derived from US currency and financial hegemony and is a vital source of US hegemony itself, not some optional add-on. Any announcement of America's exit from hegemony with its financial linchpin intact must remain premature.

## Conclusion

Midway through the first year of the COVID-19 pandemic, the global hegemon has more cases and more new cases per capita than any other liberal democracy. This article has analysed the global pandemic's impact on the United States’ hegemonic position by conceptualizing COVID-19 as a ‘public bad’. I argue that, owing to two differences between the COVID-19 public bad and other public bads such as climate change mitigation, and public goods such as free trade, the failure to adequately manage the COVID-19 public bad could have more far-reaching consequences for US hegemony. The COVID-19 public bad, like other public bads and goods, can be evaluated according to their degree of rivalry and excludability. But, unlike climate change mitigation, and many of the most important public goods that make up the LIO—international security, open trade and finance, and the dollar system—the COVID-19 public bad not only has some degree of rivalry but can be made excludable, transforming it into more of a club good. These properties require states either to tackle the COVID-19 public bad or to face extremely negative repercussions.

The global hegemon failed to manage the public bad, treating it as more familiar types of international relations problems where the costs of failed public bad prevention or public good provision could be externalized onto other states. With COVID-19, however, the US can only free ride on other states economically, and even so, only to the extent that the public health crisis does not crowd out the relative economic advantage of eschewing public bad prevention. Failed COVID-19 leadership has entrenched the public bad problem in the US, jeopardizing the domestic foundations of US hegemony—a population free from existential threats and a healthy economy. This could in turn threaten America's capacity to provide global public goods. At the same time, the US government has strategically used the public bad to undermine America's commitment to relatively open borders. Though not central to the LIO, free movement of people is one of its dimensions.

Policy coordination failure and US withdrawal from the WHO will also make the US more vulnerable to health crises, since US health experts will miss out on information exchange within the institution. Combined with the reduction in funding for the CDC, withdrawal from the WHO represents an abdication of US leadership in the face of global health crises, and could make it harder for the US to manage COVID-19 and future public health bads. However, while US withdrawal is in itself regrettable, the ramifications for US hegemony as a result of this particular decision are unlikely to be serious.

It is, in fact, in the least institutionalized component of the LIO, the provision of macroeconomic stability through US monetary and currency leadership, that the United States has exercised clear, decisive leadership. As banker to the world, the US Federal Reserve has acted as ‘lender of last resort’, extending emergency dollar funding to foreign central banks in order to stabilize asset markets and foreign currencies, as theories of hegemony predict. Could the United States extend its hegemony by specializing in monetary, currency and financial leadership? Absolutely. But the key point to take away from the above analysis is that the COVID-19 public bad could ricochet back on US monetary hegemony by eroding its economic foundations, eventually compromising the Federal Reserve's capacity to stabilize the global economy by acting as lender of last resort.

Yet, in order for the United States to lose its pre-eminent global position, an alternative to American power has to come into view. To date, as we watch the global spread of the pandemic, the available evidence suggests that COVID-19 has hit the interdependent open liberal democracies especially hard, placing question marks against liberal alternatives to US hegemony. That leaves China or Russia, both of which have been hard hit by the pandemic, both in real terms and in terms of their legitimacy. Moreover, neither China nor Russia has the multidimensional wherewithal to replace US power.

As we look ahead, COVID-19 renews old debates, silenced by the intellectual orthodoxy of neo-liberal institutionalism, regarding the conditions for hegemonic decline and the relevance of international institutions.

## Footnotes

1 Christopher Layne, ‘The US–Chinese power shift and the end of Pax Americana’, *International Affairs* 94: 1, Jan. 2018, pp. 89–112; Nana de Graaff and Bastiaan van Apeldoorn, ‘US–China relations and the liberal world order: contending elites, competing visions’, *International Affairs* 94: 1, Jan. 2018, pp. 113–32; Joseph S. Nye, Jr, ‘The rise and fall of American hegemony from Wilson to Trump’, *International Affairs* 95: 1, Jan. 2019, pp. 63–80.

2 Michael Mandelbaum, *The frugal superpower: America's global leadership in a cash-strapped era* (New York: PublicAffairs, 2010).

3 Fareed Zakaria, *The post-American world* (London: Allen Lane, 2008); Parag Khanna, *The Second World: how emerging powers are redefining global competition in the twenty-first century* (New York: Random House, 2008).

4 Charles A. Kupchan, *No one's world: the West, the rising rest, and the coming global turn* (Oxford: Oxford University Press, 2012).

5 John J. Mearsheimer, ‘Bound to fail: the rise and fall of the liberal international order’, *International Security* 43: 4, 2019, pp. 7–50.

6 Yuen Foong Khong, ‘Power as prestige in world politics’, *International Affairs* 95: 1, Jan. 2019, pp. 119–42.

7 Jim O’Neill, *The growth map: economic opportunity in the BRICS and beyond* (New York: Penguin, 2011).

8 Thomas Oatley, W. Kindred Winecoff, Andrew Pennock and Sarah Bauerle Danzman, ‘The political economy of global finance: a network model’, *Perspectives on Politics* 11: 1, 2013, pp. 133–53; Carla Norrlof, ‘Dollar hegemony: a power analysis’, *Review of International Political Economy* 21: 5, 2014, pp. 1042–70; Jan Fichtner, ‘Perpetual decline or persistent dominance? Uncovering Anglo-America's true structural power in global finance’, *Review of International Studies* 43: 1, 2017, pp. 3–28; Daniel McDowell, *Brother, can you spare a billion? The United States, the IMF, and the international lender of last resort* (Oxford: Oxford University Press, 2016); Carla Norrlof, Paul Poast, Benjamin J. Cohen, Sabreena Croteau, Aashna Khanna, Daniel McDowell, Hongying Wang and W. Kindred Winecoff, ‘Global monetary order and the liberal order debate’, *International Studies Perspectives* 21: 2, 2020, pp. 109–53.

9 Carla Norrlof, ‘Raison de l'hégémonie (the hegemon's interest): theory of the costs and benefits of hegemony’, *Security Studies* 28: 3, 2019, pp. 423–50; Oatley et al., ‘The political economy of global finance’.

10 Daniel W. Drezner, ‘Immature leadership: Donald Trump and the American presidency’, *International Affairs* 96: 2, March 2020, pp. 383–400.

11 Nancy H. L. Leung, Daniel K. W. Chu, Eunice Y. C. Shiu, Kwok-Hung Chan, James J. McDevitt, Benien J. P. Hau, Hui-Ling Yen, Yuguo Li, Dennis K. M. Ip, J. S. Malik Peiris, Wing-Hong Seto, Gabriel M. Leung, Donald K. Milton and Benjamin J. Cowling, ‘Respiratory virus shedding in exhaled breath and efficacy of face masks’, *Nature Medicine*, vol. 26, 2020, pp. 676–80.

12 Joseph T. Wu, Kathy Leung, Mary Bushman, Nishant Kishore, Rene Niehus, Pablo M. de Salazar, Benjamin J. Cowling, Marc Lipsitch and Gabriel M. Leung, ‘Estimating clinical severity of COVID-19 from the transmission dynamics in Wuhan, China’, *Nature Medicine*, vol. 26, 2020, pp. 506–10.

13 Robert Verity, Lucy C. Okell, Ilaria Dorigatti, Peter Winskill, Charles Whittaker, Natsuko Imai, Gina Cuomo-Dannenburg, Hayley Thompson, Patrick G. T. Walker, Han Fu, Amy Dighte, Jamie T. Driffin, Marc Baguelin, Sangeeta Bhatia, Adhiratha Boonyasiri, Anne Cori, Zulma Cucunubá, Rich FitzJohn, Katy Gaythorpe, Will Green, Arran Hamlet, Wes Hinsley, Daniel Laydon, Gemma Nedjati-Gilani, Steven Riley, Sabine van Elsland, Erik Volz, Haowei Want, Yuanrong Wang, Xiaoyue Xi, Christl A. Donnelly, Azra C. Ghani and Neil M. Ferguson, ‘Estimates of the severity of Coronavirus Disease 2019: a model-based analysis’, *Lancet Infectious Diseases*, 3 April 2020, p. 7.

14 Jiao Zhao, Yan Yang, Hanping Huang, Dong Li, Dongfeng Gu, Xiangfeng Lu, Zheng Zhang, Lei Liu, Ting Liu, Yukun Liu, Yunjiao He, Bin Sun, Meilan Wei, Guangyu Yang, Xinghuan Wang, Li Zhang, Xiaoyang Zhou, Mingzhao Xing and Peng George Wang, ‘Relationship between the ABO blood group and the COVID-19 susceptibility’, medRxiv, 27 March 2020; Peter Arend, ‘How blood group A might be a risk and blood group O be protected from coronavirus (COVID-19) infections (how the virus invades the human via blood group carbohydrate)’, medRxiv, 6 May 2020, pp. 1–18.

15 Wu et al., ‘Estimating clinical severity of COVID-19’.

16 Note that these findings are based on a small sample: see Michael Day, ‘COVID-19: four fifths of cases are asymptomatic, China figures indicate’, *British Medical Journal*, BMJ369:m1375, 2 April 2020.

17 Freedom House, ‘Global Freedom Scores’ (Washington DC, 2020).

18 Michael Coppedge et al., *V-Dem Dataset V10* (Gothenburg: Varieties of Democracy (V-Dem) Project, 2020).

19 Todd Sandler, *Global challenges: an approach to environmental, political, and economic problems* (Cambridge and New York: Cambridge University Press, 1997), p. 11.

20 Carla Norrlof, ‘Hegemony and inequality’, *International Affairs* 94: 1, Jan. 2018, pp. 63–88.

21 John Gerard Ruggie, ‘International regimes, transactions, and change: embedded liberalism in the postwar economic order’, *International Organization* 36: 2, 1982, pp. 379–415.

22 Yasmeen Abutaleb, Josh Dawsey, Ellen Nakashima and Greg Miller, ‘The US was beset by denial and dysfunction as the coronavirus raged’, *Washington Post*, 4 April 2020.

23 Shane Harris, Greg Miller, Josh Dawsey and Ellen Nakashima, ‘US intelligence reports from January and February warned about a likely pandemic’, *Washington Post*, 21 March 2020.

24 WHO, ‘Statement on the Second Meeting of the International Health Regulations (2005) Emergency Committee regarding the outbreak of novel coronavirus (2019-Ncov)’ (Geneva, 30 Jan. 2020).

25 WHO, ‘Statement on the Second Meeting of the International Health Regulations (2005) Emergency Committee’.

26 Matthew J. Belvedere, ‘Trump says he trusts China's Xi on coronavirus and the US has it “totally under control”’, *CNBC*, 22 Jan. 2020.

27 Maggie Haberman, ‘Trade adviser warned White House in January of risks of a pandemic’, *New York Times*, 6 April 2020.

28 Aaron Rupar, ‘Trump said “nobody could have predicted” coronavirus. White House memos show his advisers did’, *Vox*, 7 April 2020.

29 Eric Lipton, David E. Sanger, Maggie Haberman, Michael D. Shear, Mark Mazzetti and Julian E. Barnes, ‘He could have seen what was coming: behind Trump's failure on the virus’, *New York Times*, 11 April 2020.

30 Lipton et al., ‘He could have seen what was coming’.

31 CDC, ‘Transcript for the CDC telebriefing update on COVID-19’ (Washington DC, 26 Feb. 2020).

32 The White House, ‘Remarks by President Trump, Vice President Pence, and members of the Coronavirus Task Force in press conference’, James S. Brady Press Briefing Room (Washington DC, 27 Feb. 2020).

33 The White House, ‘Remarks’, 27 Feb. 2020.

34 Donald J. Trump, ‘Proclamation on declaring a national emergency concerning the novel coronavirus disease (COVID-19) outbreak’ (Washington DC: The White House, 13 March 2020).

35 Donald J. Trump, ‘Executive order on national emergency authority to temporarily extend deadlines for certain estimated payments’ (Washington DC: The White House, 13 March 2020).

36 Donald J. Trump, ‘The president's coronavirus guidelines for America’ (Washington DC: The White House, 16 March 2020).

37 Mark Mazzetti, Julian E. Barnes, Edward Wong and Adam Goldman, ‘Trump officials are said to press spies to link virus and Wuhan labs’, *New York Times*, 30 April 2020.

38 Peng Zhou, Xing-Lou Yang, Xian-Guang Wang, Ben Hu, Lei Zhang, Wei Zhang, Hao-Rui Si, Yan Zhu, Bei Li, Chao-Lin Huang, Hui-Dong Chen, Jing Chen, Yun Luo, Hua Guo, Ren-Di Jiang, Mei-Qin Liu, Ying Chen, Xu-Rui Shen, Xi Wang, Xiao-Shuang Zheng, Kai Zhao, Quan-Jiao Chen, Fei Deng, Lin-Lin Liu, Bing Yan, Fa-Xian Zhan, Yan-Yi Wang, Geng-Fu Xiao and Zheng-Li Shi, ‘A pneumonia outbreak associated with a new coronavirus of probable bat origin’, *Nature* 579: 7798, 2020, pp. 270–73.

39 Zoonotic transfer is an infection transmitted from an animal to a human host. See Kristian G. Andersen, Andrew Rambaut, W. Ian Lipkin, Edward C. Holmes and Robert F. Garry, ‘The proximal origin of Sars-Cov-2’, *Nature Medicine* 26: 4, 2020, pp. 450–52.

40 Andersen et al., ‘The proximal origin of Sars-Cov-2’.

41 Andersen et al., ‘The proximal origin of Sars-Cov-2’, p. 450.

42 Black Knight, ‘May 2020 report’, Mortgage Monitor, May 2020, p. 16.

43 Ayşegül Şahin, Murat Tascı and Jın Yan, ‘The unemployment cost of COVID-19: how high and how long?’, *Economic Commentary* (Cleveland, OH: Federal Reserve Bank of Cleveland, 2020).

44 The two main elements within this are the Payroll Protection Program and the Economic Injury Disaster Loan Program.

45 US Treasury, ‘The CARES Act works for all Americans’ (Washington DC, 27 March 2020).

46 Robin Saks Frankel, ‘The Senate passed another coronavirus relief bill. What's in it for small businesses?’, *Forbes*, 22 April 2020.

47 SBA, ‘Coronavirus (COVID-19): small business guidance and loan resources’ (Washington DC, 2020).

48 Board of Governors of the Federal Reserve System (BGFRS), ‘Federal Reserve issues FOMC statement’ (Washington DC, 15 March 2020).

49 BGFRS, ‘Federal Reserve announces extensive new measures to support the economy’ (Washington DC, 23 March 2020).

50 BGFRS, ‘Federal Reserve announces extensive new measures’.

51 BGFRS, ‘Federal Reserve announces extensive new measures’.

52 Jessica Semega, Melissa Kollar, John Creasmer and Abinash Mohanty, ‘Income and poverty in the United States: 2018’ (Washington DC: US Census Bureau, 20 Sept. 2019).

53 Edward R. Berchick, Jessica C. Barnett and Rachel D. Upton, ‘Health insurance coverage in the United States: 2018’ (Washington DC: US Census Bureau, 8 Nov. 2019).

54 Sonam Sheth and Gina Heeb, ‘Trump spent the past 2 years slashing the government agencies responsible for handling the coronavirus outbreak’, *Business Insider*, 25 Feb. 2020; Laurie Garrett, ‘Trump has sabotaged America's coronavirus response’, *Foreign Policy*, 31 Jan. 2020.

55 Zolan Kanno-Youngs, ‘What's in President Trump's fiscal 2021 budget?’, *New York Times*, 10 Feb. 2020.

56 Karyn Schwartz, ‘Coronavirus response and the Affordable Care Act’ (San Francisco: Kaiser Family Foundation, 23 March 2020).

57 Schwartz, ‘Coronavirus response and the Affordable Care Act’.

58 Donald J. Trump, ‘Proclamation—suspension of entry as immigrants and nonimmigrants of certain additional persons who pose a risk of transmitting 2019 novel coronavirus’ (Washington DC: The White House, 11 March 2020).

59 The White House, ‘Remarks by President Trump, Vice President Pence, and members of the Coronavirus Task Force in Press Conference’, James S. Brady Press Briefing Room (Washington DC: The White House, 29 Feb. 2020).

60 The White House, ‘Remarks’, 29 Feb. 2020.

61 Department of Homeland Security (DHS), ‘Joint statement on US–Canada joint initiative: temporary restriction of travelers crossing the US–Canada land border for non-essential purposes’ (Washington DC, 2020); DHS, ‘Joint statement on US–Mexico joint initiative to combat the COVID-19 pandemic’ (Washington DC, 20 March 2020).

62 Kanno-Youngs, ‘What's in President Trump's fiscal 2021 budget?’.

63 The White House, ‘President Donald J. Trump is honoring his commitment to protect American workers by temporarily pausing immigration’ (Washington DC, 2020); The White House, ‘Proclamation suspending entry of aliens who present a risk to the US labor market following the coronavirus outbreak’ (Washington DC, 22 April 2020).

64 The White House, *The National Security Strategy of the United States of America* (Washington DC, 2017).

65 ‘Trump says he'll “put a hold” on World Health Organization funding before backtracking’, *Time*, 7 April 2020.

66 Patrice A. Harris, ‘AMA statement on halting World Health Organization funding’ (Chicago: American Medical Association, 14 April 2020).

67 Nancy Pelosi, ‘Statement on President Trump halting WHO funding’ (Washington, DC, 15 April 2020).

68 Pien Huang, ‘Trump and WHO: how much does the US give? What's the impact of a halt in funding?’, National Public Radio, 15 April 2020.

69 G. John Ikenberry, *Liberal Leviathan: the origins, crisis, and transformation of the American world order* (Princeton: Princeton University Press, 2011). See also David A. Lake, *Hierarchy in international relations* (Ithaca, NY: Cornell University Press, 2009).

70 Charles P. Kindleberger, ‘Dominance and leadership in the international economy’, *International Studies Quarterly* 25: 2, 1981, pp. 198–200.

71 Ben S. Bernanke, *The Federal Reserve and the financial crisis* (Princeton: Princeton University Press, 2013); McDowell, *Brother, can you spare a billion?*.

72 Norrlof, ‘Dollar hegemony’.

73 Susan Strange, ‘International economics and international relations: a case of mutual neglect’, *International Affairs* 46: 2, 1970, pp. 304–15.

74 Daniel McDowell, ‘The US as “sovereign international last-resort lender”: the Fed's currency swap programme during the great panic of 2007–09’, *New Political Economy* 17: 2, 2012, pp. 157–78.

75 BGFRS, ‘Coordinated central bank action to enhance the provision of US dollar liquidity’ (Washington DC, 15 March 2020).

76 BGFRS, ‘Federal Reserve announces the establishment of temporary US dollar liquidity arrangements with other central banks’ (Washington DC, 19 March 2020).

77 Catharina J. Hooyman, ‘The use of foreign exchange swaps by central banks’, *Staff Papers (International Monetary Fund)* 41: 1, 1994, p. 91.

78 Michael D. Bordo, Owen F. Humpage and Anna J. Schwartz, *The evolution of the Federal Reserve swap lines since 1962*, NBER working paper no. 20755 (Cambridge, MA: National Bureau of Economic Research, 2014).

79 The White House, ‘Remarks by President Trump, Vice President Pence, and members of the Coronavirus Task Force in press briefing’, Oval Office (Washington DC, 15 March 2020).

